# Assessment of the willingness of Nigerian Orthodontists to offer face-to-face orthodontic services to patients infected with Covid-19

**DOI:** 10.4314/ahs.v23i1.7

**Published:** 2023-03

**Authors:** Tope Emmanuel Adeyemi, Monica Ndudi Adekoya, Elfleda Angelina Aikins

**Affiliations:** 1 Department of Child Dental Health, Faculty of Dentistry, Bayero University, Kano/ Aminu Kano Teaching Hospital, Kano; 2 Department of Child Dental Health, University of Calabar/ University of Calabar Teaching Hospital; 3 Department of Child Dental Health, College of Health Sciences, University of Port Harcourt/ University of Port Harcourt Teaching Hospital

**Keywords:** Emergency orthodontics, COVID-19, unwillingness to treat, trauma, aligners

## Abstract

**Introduction:**

Efforts to contain the spread of the COVID-19 disease led to suspension of many services which caused orthodontists to resort to providing only emergency services. The aims of this study were to assess the willingness of Nigerian Orthodontists and Orthodontic resident doctors to treat patients infected with Covid-19 as well as to ascertain advice given regarding traumatic arch wires and aligners during the pandemic.

**Participants and study design:**

This was a descriptive cross-sectional study. We included dentists who were Orthodontists and orthodontic resident doctors practicing in Nigeria. Data was collected via an online questionnaire (Google form) which was sent three times weekly throughout May/June, 2020 and analysed using SPSS version 23. Descriptive statistics were used to analyse the data.

**Results:**

Forty-eight out of the 90 members of the WhatsApp group responded within the allotted time, giving a response rate of 53.3%. Out of a total of 48 respondents, 39 (81.3%) were unwilling to treat patients infected with COVID-19 during the pandemic. The major reason was fear of infection with the virus (12, 25.0%). Most of the respondents (41, 55.4%) suggested the use of wax for relief of trauma from arch wires.

**Conclusion:**

Majority of Orthodontists and orthodontic residents practicing in Nigeria expressed unwillingness to treat patients infected with COVID-19. Aligner therapy was proffered as an alternative to bracket therapy.

## Introduction

From the beginning of 2020, coronavirus disease 2019 (COVID-19) caused by severe acute respiratory syndrome coronavirus 2 (SARS-CoV-2) spread throughout the world causing a major upheaval.^1^ Efforts to contain the spread of the disease led to the suspension of many services which included orthodontic treatment.^1^ This scenario caused anxiety for many orthodontic patients as well as orthodontists as only emergency services were provided at this time.^2^

As the pandemic progressed, orthodontists sought novel ways of communicating with their patients. Social media handles especially WhatsApp became very useful communication tools.^3^ The ability to communicate remotely served both to allay the anxieties of patients and to enable the monitoring of progression of treatment. Tele-orthodontics became a popular tool during the pandemic and still remains relevant in order to ease treatment demands on the Orthodontist as well as the patient without reducing the quality of orthodontic treatment received.^4^

Although the pandemic has been brought somewhat under control with effective treatments and vaccinations and orthodontic offices having reopened, there is still a lot of anxiety among Orthodontists due to the uncertain nature of the disease and the ease with which the disease can be contracted in the dental office. This anxiety has impacted the attitude of Orthodontists towards their practice as well as Orthodontist-patient relationships.^5^ Many Orthodontists did not resume full practice immediately due to the fore mentioned factors and continued with remote consultations via WhatsApp platforms and other social media handles. Essential areas of adaptation to ensure in-person safe practice of orthodontics included “microbiologic control measures, social distancing, new ergonomics and bioethical considerations”.^3^

Covid-19 is a disease with variable manifestations whereby some patients do not develop symptoms although they are infected with the coronavirus and can transmit the infection. We therefore thought it expedient to report on the willingness of Nigerian Orthodontists and Orthodontic resident doctors to treat patients infected with COVID-19 as well as to ascertain advice given to orthodontic patients regarding traumatic arch-wires and aligners during this period.

## Materials and Methods

Ethical approval from the Ethics Committee of the Aminu Kano Teaching Hospital, Kano, Nigeria, and informed consent from each participant were obtained before the commencement of this descriptive cross-sectional study.

Participants: This study was carried out among Orthodontists and orthodontic resident doctors practicing in Nigeria. We included Dentists who were either Orthodontists or Residents doctors who were in training to become Orthodontists.

### Setting

This was carried out in Nigeria.

### Design

This was a cross-sectional survey. Data was collected via an online questionnaire (Google form) which was validated by testing the questionnaire on three consultants and two resident doctors with suggested corrections made before it was sent to the WhatsApp group containing dentists who met the inclusion criteria. Reminders were sent to the WhatsApp group and also to the individual WhatsApp pages of eligible participants three times weekly throughout May/June, 2020.

The questionnaire was made up of two sections. Section A sought information concerning Socio-demographics, section B assessed the participant's willingness to treat patients infected with COVID-19; reasons for lack of willingness, advice given concerning orthodontic emergencies and their opinion concerning the use of aligners as it relates to COVID-19. The responses to the questionnaires were downloaded into a passworded computer for data protection purposes and then analysed using IBM SPSS Statistics for Windows, Version 23.0. Armonk, NY: IBM Corp. Descriptive statistics were used to analyse the data.

## Results

Out of the ninety orthodontists and orthodontic residents on the WhatsApp group, forty-eight of them responded within the allotted time, giving a response rate of 53.3%.

### Socio-demographics

As seen from the data presented in Table 1, most of the Orthodontists were relatively young and middle-aged. Twenty respondents were aged 31-40 years accounting for 20 (41.7%) of the responses. This was closely followed by those aged 41-50 years making up 19 (39.6%) of the study population.

**Table 1:** Sociodemographic characteristics of participants

Variable	Frequency (48)	Percent (100)
Age:		
20–30 years	2	4.2
31–40 years	20	41.7
41–50 years	19	39.6
51–60 years	5	10.4
61–70 years	2	4.2
Gender		
Male	19	39.6
Female	29	60.4
Years of practice		
< 10 years	6	12.5
10 - 19 years	26	54.2
20 - 29 years	13	27.1
30 - 39 years	3	6.3
Region of Practice		
Lagos	24	50.0
North	5	10.4
South	19	39.6
Professional Status		
Junior resident	8	16.7
Senior resident	18	37.5
Specialist/Consultant	22	45.8
Type of practice		
Government/ Military hospital	39	81.3
Private practice	3	6.3
Private practice, Government/ Military hospital	6	12.5

Twenty-four (50.0%) orthodontists were practicing in Lagos, 19 (39.6%) in Southern Nigeria and 5 (10.4%) in Northern Nigeria. The gender demographics indicate that there were more female orthodontists than males as evidenced by 29 (60.4%) of the responses being female. A greater number of responses 26 (54.2%) were received from those who had practiced 10-19 years. Regarding the respondents' practice status, 22 (45.8%) were specialists/consultants; 18 (37.5%) were senior residents; and 8 (16.7%) were junior residents. Most (39, 81.3%) of the orthodontists practiced in government or military hospitals.

### Clinical details

**Figure 1:**
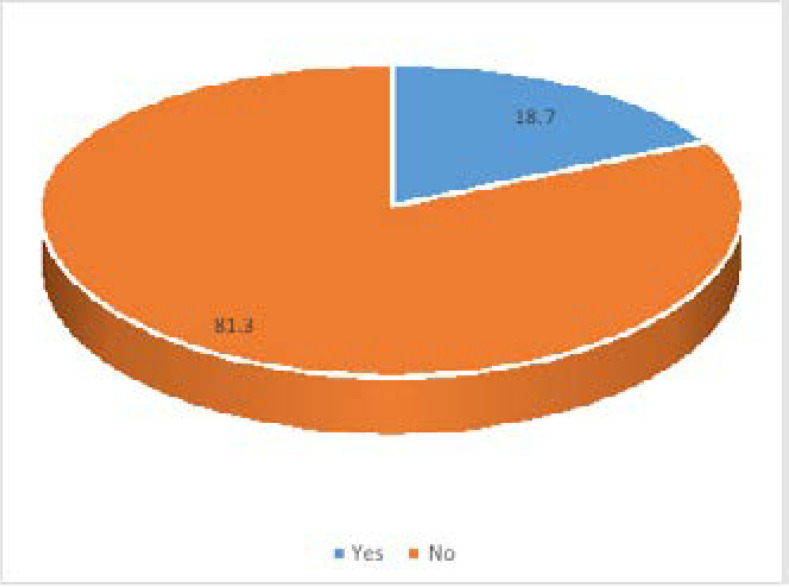
Participants willingness to offer face-to-face orthodontic services to patients infected with COVID-19

Most of the orthodontists (39, 81.3%) were unwilling to treat patients infected with COVID-19 face-to-face during the pandemic as seen in Fig 1. Over a quarter (14, 29.2%) of the respondents did not give any reason for this. The major reason cited was largely as a result of fear of infection with the virus (12, 25.0%). Other reasons were lack of Personal Protective Equipment (PPE) (6, 12.5%), highly pathogenic nature of the virus (2, 4.2%), orthodontic treatment not considered an absolute emergency (2, 4.2%), and that COVID 19 patients should be in isolation centers (2, 4.2%). Table 2

**Table 2:** Participants' willingness to offer face-to-face orthodontic services to patients infected with Covid-19

Reason	Frequency	Percent
High risk	1	2.1
Orthodontic care can always wait.	1	2.1
Patient will be highly contagious.	1	2.1
Can easily get infected by aerosols from the patients	1	2.1
Cost and availability of N95 masks	1	2.1
I would rather wait for the patient to test negative, except in orthodontic emergency cases	1	2.1
It is highly contagious, no distancing when treating patients	1	2.1
it is highly transmissible	1	2.1
Only treat with necessary PPE	1	2.1
Personal	1	2.1
Treatment of the COVID-19 should be of priority coupled with the fact that the mode of spread of the virus is not yet fully understood	1	2.1
Highly pathogenic virus	2	4.2
Orthodontic treatment not an absolute emergency	2	4.2
Patient should be in isolation centre	2	4.2
Lack of PPEs	6	12.5
Fear of infection	12	25.0
No response (Did not indicate any reason for their unwillingness)	14	29.2

When asked to give advice to patients with traumatic wires during the pandemic, most of the orthodontists (41, 55.4%) suggested the use of wax for temporary relief. Thirty (40.4%) suggested the use of a small nail clipper to trim the wire. Other suggestions were use of small wire cutter (1,1.4%), use of pencil eraser to tuck it back (1,1.4%); and calling the clinic (1,1.4%). These data are presented in Table 3.

**Table 3:** Advice given to patients with traumatic wires during the pandemic

Advice	Frequency	Percent
Use small wire cutter	1	1.4
Use pencil eraser	1	1.4
Call the clinic	1	1.4
Use a small nail cutter	30	40.4
Use orthodontic wax	41	55.4

The participants' opinions about the usage of aligners during the pandemic, are depicted in table 4. Thirty (62.5%) agreed to the usage of the current aligner by patients infected with COVID-19 while 11 (22.9%) disagreed. Twenty-two (45.8%) participants disagreed that aligner posed a risk of re-infecting patients. Regarding the risk to clinicians, 28 (58.3%) opined that the risk was lower among orthodontist than general dentists while 20, (41.7%) opined that the risk was higher for orthodontists.

**Table 4:** Participants' opinions of usage of aligners during the pandemic

Variable	Yes N/%	No N/%	I Don't know N/%
Utilization of current aligner if infected.	30 (62.5%)	11 (22.9%)	7 (14.6%)
Aligners pose a risk of reinfecting patients.	16 (33.3%)	22 (45.8%)	10 (20.8%)
Orthodontists are at higher risk of COVID-19 infection than general Dentists.	20 (41.7%)	28 (58.3%)	0 (0.0)

## Discussion

This study showed that majority of the Orthodontists/Orthodontic residents practicing in Nigeria were unwilling to offer face-to-face orthodontic services to patients infected with COVID-19 during the pandemic. This is similar to the findings from a study carried out previously.^6^ Although some of the respondents did not give any reason for their unwillingness to treat such patients, others cited fear of infection with the virus, lack of Personal Protective Equipment (PPE), the highly pathogenic nature of the virus, orthodontic treatment not being considered an absolute emergency by them and that patients infected with COVID-19 should be in isolation centers receiving treatment for the ailment. Anxiety and fear concerning infection with the virus among dental practitioners was also found to be high in Italy where almost 85% of participants were worried about contracting the virus whilst attending to patients.^7^ In contrast to our findings concerning the willingness to offer face-to-face treatment to patients infected with Covid-19, reports from a study by Ottolenghi et al in New York revealed that only about 8.9% of health workers were unwilling to treat patients infected with SAR-Cov-2.^8^ Another study by Rafi et al, in Bangladesh, revealed that over 69% of physicians were willing to work during the Covid-19 pandemic.^9^ This could be due to availability of personal protective equipment, support to maintain recommended quarantine and isolation policy and adequate and effective training of staff.^9^

A previous study^10^ has documented the fact that females seem to be more willing to participate in research than males but in our study, the demographics revealed female preponderance among the participants which is reflective of the fact that there were more females in the WhatsApp group.

The unwillingness to treat such patients infected with COVID-19 exhibited by our participants may also be due to the fact that about half of them reside and practice in Lagos State which was the epicentre of the COVID-19 outbreak in the country at the period of conducting this study. They may have had firsthand experience in terms of the presentation and complications of COVID-19, thus leading to fear and anxiety in them as reported by another Nigerian study.^11^ In a Chinese study carried out among orthodontists, orthodontic residents and nurses, about two-thirds of the population were found to be unwilling to treat patients infected with COVID-19 which is lower than the proportion in our study, however their reasons for this behaviour were largely the same.^12^ The fear of infection which may lead to infection of their family members was their major reason which was the same reason cited by most of the Chinese participants as well as dentists from 30 countries worldwide.^12^,^13^

The risk of transmission of COVID-19 is high among those who are in close proximity to infected individuals.^14^ Orthodontists and orthodontic resident doctors work in close contact with patients and vice versa. Orthodontic procedures require that the orthodontist be in close proximity to the patient over a considerable length of time (working field and the dentist is averagely 35-40 cm)^15^ which therefore puts both the orthodontists and their patients at great risk of cross infection when either of them happens to be infected with the virus.^1^ The participants perceived lower risk of infection among orthodontists as compared to general dentists, may be because the latter engage in more aerosol generating procedures than orthodontists.

During the period of lockdown, orthodontists were attending exclusively to orthodontic emergencies. Orthodontic emergencies during the pandemic referred to problems with aligners, traumatic wires as well as loose brackets and bands.^16^ Majority of problems encountered by our patients during this period were related to traumatic wires which is similar to that seen in another Nigerian study^17^ and in a Saudi Arabian study^18^ but was different in other studies where bracket, archwire, molar band and tubes breakage were the most encountered emergencies.^19^,^20^,^21^ These problems were communicated via online platforms as advised by other authors.^16^ A study^22^ (YANA) in the United Kingdom revealed that video and telephone consultations were the means used to communicate with their patients during the pandemic. Our patients were encouraged to use wax for temporary relief, small nail clippers or wire cutters to trim the wire, or use a pencil eraser to tuck it back. This advice is similar to what obtained in other parts of the world.^2^,^19^,^23^.

Only a few of the participants indicated that they would advise the patients to call the clinic. This is in agreement with recommendations from previous studies on the management of orthodontic emergencies during 2019-NCOV which in addition to the suggestions above, also recommended that patients could carefully try to remove the arch wire with eyebrow tweezers. It is important to promptly resolve these orthodontic urgencies as quickly as possible in order to prevent decreasing the motivation of the patient, losing the patients' confidence in the orthodontist and to provide relief from pain and discomfort for the patient.^16^,^19^,^24^.

Aligner therapy has been suggested to be one method of enabling orthodontic treatment to continue without constant contact with the orthodontist, especially during this pandemic.^25^,^26^ It was noted in a Chinese study that during the pandemic, patients on aligner therapy had less complaints/emergencies than those using fixed appliances.^27^ Also the patients using fixed appliances were more anxious during the pandemic.^27^ Aligners may be used in conjunction with fixed Orthodontic appliances at the beginning or end of treatment. Our study shows that most of the participants agreed to the usage of the current aligner by patients infected with COVID-19. This is in agreement with recommendations from a study by Alberto Caprioglio et al^10^ as well as the Chinese study^27^ in which it was suggested that patients remained on their current aligner until the end of the emergency. It was also suggested that in the case of a broken aligner, patients should use the previous aligner or they should change to the next one depending on the duration of usage of the broken/lost aligner.^16^

One limitation of this study is the low response rate of 53.3%. This is attributable to burn out in filling various online questionnaires by different researchers which took place during the lock down period.

The implication of this study for clinical practice is the fact that patients could and should be taught on how to handle traumatic wires at home during normal consultation so that when the need to use it arises and the Orthodontist is not available due to unforeseen circumstance such as lockdown as occasioned by COVID-19 pandemic, such patients will then be able to render help for themselves easily.

One of the implications of this study for research is the need to assess the impact of the COVID-19 pandemic on the time taken to complete an orthodontic case.

## Conclusion

Majority of the orthodontists and orthodontic residents practicing in Nigeria expressed unwillingness to offer face-to-face orthodontic treatment to patients infected with COVID-19. The main reason for this was their fear of being infected by the virus and infecting family members with same.

They preferred to communicate with their patients via online platforms and proffer temporary solutions to traumatic wires. These solutions included the use of orthodontic wax and a small nail cutter to cut these traumatic wires. Most participants advised their patients to continue to use their current aligners.

## References

[1] Suri S, Vandersluis Y, Kochhar A, Bhasin R, Abdallah M (2020). Clinical orthodontic management during the COVID-19 pandemic. Angle Orthod.

[2] Yilmaz H, Ozbilen E (2020). The Assessment of Knowledge, Behaviors, and Anxiety Levels of the Orthodontists about COVID-19 Pandemic. Turk J Orthod.

[3] Garcia-Camba P, Marcianes M, Morales M (2020). Changes in orthodontics during the COVID-19 pandemic that have come to stay. Am J Orthod Dentofac Orthop.

[4] Saccomanno S, Quinzi V, Sarhan S, Laganà D, Marzo G (2020). Perspectives of tele-orthodontics in the COVID-19 emergency and as a future tool in daily practice. Eur J Paediatr Dent.

[5] Lindauer S (2020). COVID-19 affecting our world. Angle Orthod.

[6] Isiekwe IG, Adeyemi ET, Aikins EA (2021). The COVID-19 pandemic and orthodontic practice in Nigeria. J Orthod Sci.

[7] Consolo U, Bellini P, Bencivenni D, Iani C, Checchi V (2020). Aspects and Psychological Reactions to COVID-19 of Dental Practitioners in the Northern Italy Districts of Modena and Reggio Emilia. Int J Env Res Public Heal.

[8] Ottolenghi J, Mclaren RA, Bahamon C, Dalloul M, McCalla S, Minkoff H (2021). Health care workers' attitude towards patients with COVID-19. Open Forum Infect Dis.

[9] Rafi MA, Hasan MT, Azad DT, Alam ST, Podder V, Hossain S (2021). Willingness to work during initial lockdown due to COVID-19 pandemic. Study based on an online survey among physicians of Bangladesh. PLos ONE.

[10] Qiu J, Shen B, Zhao M, Wang Z, Xie B, Xu Y (2020). A nationwide survey of psychological distress among Chinese people in the COVID-19 epidemic: implications and policy recommendations. Gen Psychiatr.

[11] Olabintan AA, Otuyemi OD, Adeyemi TE, Soyoye OA, Sanu OO Perceived fear of Covid-19 infection among Nigerian dental health care workers. Tropical Dental Journal.

[12] Hua F, Qin D, Yan J, Zhao T, He H (2020). COVID-19 Related Experience, Knowledge, Attitude, and Behaviors Among 2,669 Orthodontists, Orthodontic Residents, and Nurses in China: A Cross-Sectional Survey. Front Med (Lausanne).

[13] Ahmed MA, Jouhar R, Ahmed N (2020). Fear and Practice Modifications among Dentists to Combat Novel Coronavirus Disease (COVID-19) Outbreak. Int J Environ Res Public Health.

[14] Peng X, Xu X, Li Y, Cheng L, Zhou X, Ren B (2020). Transmission routes of 2019-nCoV and controls in dental practice. Int J Oral Sci.

[15] Pîrvu C, Pătraşcu I, Pîrvu D, Ionescu C (2014). The dentist's operating posture - ergonomic aspects. J Med Life.

[16] Caprioglio A, Pizzetti GB, Zecca PA, Fastuca R, Maino G, Nanda R (2020). Management of orthodontic emergencies during 2019-NCOV. Prog Orthod.

[17] Isiekwe IG, Adeyemi TE, Aikins EA, Umeh OD (2020). Perceived impact of the COVID-19 pandemic on orthodontic practice by orthodontists and orthodontic residents in Nigeria. J World Fed Orthod.

[18] Turkistani KA (2020). Impact of delayed orthodontic care during COVID-19 pandemic: Emergency, disability, and pain. J World Fed Orthod.

[19] Cotrin P, Peloso RM, Pini NIP, Oliveira RC, de Oliveira RCG, Valarelli FP, Freitas KMS (2020). Urgencies and emergencies in orthodontics during the coronavirus disease 2019 pandemic: Brazilian orthodontists' experience. Am J Orthod Dentofacial Orthop.

[20] Sella Tunis T, Ratson T, Matalon S, Abba M, Abramson A, Davidovitch M, Shpack N (2022). The Impact of the COVID-19 Pandemic on Israeli Orthodontic Practice: A Clinic's Activity and Patients' Attitudes. Int J Environ Res Public Health.

[21] Cotrin P, Peloso RM, Pini NIP, Oliveira RC, de Oliveira RCG, Valarelli FP, Freitas KMS (2020). Urgencies and emergencies in orthodontics during the coronavirus disease 2019 pandemic: Brazilian orthodontists' experience. Am J Orthod Dentofacial Orthop.

[22] Sabbagh Y, Lewis BR, Chadwick SM, Abu Alhaija ES (2022). The COVID-19 experience of orthodontists in the UK. J Orthod.

[23] Malekshoar M, Malekshoar M, Javanshir B (2021). Challenges, limitations, and solutions for orthodontists during the coronavirus pandemic: A review. Am J Orthod Dentofac Orthop.

[24] Dowsing P, Murray A, Sandler J (2015). Emergencies in orthodontics. Part 1: management of general orthodontic problems as well as common problems with fixed appliances. Dent Update. Am J Orthod Dentofac Orthop.

[25] Marya A, Venugopal A, Vaid N, Alam M, Karobari M (2020). Essential Attributes of Clear Aligner Therapy in terms of Appliance Configuration, Hygiene, and Pain Levels during the Pandemic: A Brief Review. Pain Res Manag.

[26] Magdalena S-D, Hanna B-V, B'nska Agata, W'zniak Krzysztof (2021). The Implications of the sCOVID-19 Pandemic on the Interest inOrthodontic Treatment and Perspectives for the Future.Real-Time Surveillance Using Google Trends. Int J Environ Res Public Heal.

[27] Feiyang G, Bojun T, Danchen Q, Tingting Z, Yuxiong S, Colman M (2020). The Impact of the COVID-19 Epidemic on Orthodontic Patients in China: An Analysis of Posts on Weibo Frontiers in Medicine. Front Med.

